# Comparative lung toxicity of engineered nanomaterials utilizing *in vitro*, *ex vivo* and *in vivo* approaches

**DOI:** 10.1186/s12951-014-0047-3

**Published:** 2014-11-26

**Authors:** Yong Ho Kim, Elizabeth Boykin, Tina Stevens, Katelyn Lavrich, M Ian Gilmour

**Affiliations:** Curriculum in Toxicology, University of North Carolina at Chapel Hill, Chapel Hill, NC USA; Environmental Public Health Division, National Health and Environmental Effects Research Laboratory, United States Environmental Protection Agency, Research Triangle Park, NC USA; Research Triangle Park Division, National Center for Environmental Assessment, United States Environmental Protection Agency, Research Triangle Park, NC USA

**Keywords:** Engineered nanomaterials, Lung toxicity, Alternative toxicity testing

## Abstract

**Background:**

Although engineered nanomaterials (ENM) are currently regulated either in the context of a new chemical, or as a new use of an existing chemical, hazard assessment is still to a large extent reliant on information from historical toxicity studies of the parent compound, and may not take into account special properties related to the small size and high surface area of ENM. While it is important to properly screen and predict the potential toxicity of ENM, there is also concern that current toxicity tests will require even heavier use of experimental animals, and reliable alternatives should be developed and validated. Here we assessed the comparative respiratory toxicity of ENM in three different methods which employed *in vivo*, *in vitro* and *ex vivo* toxicity testing approaches.

**Methods:**

Toxicity of five ENM (SiO_2_ (10), CeO_2_ (23), CeO_2_ (88), TiO_2_ (10), and TiO_2_ (200); parentheses indicate average ENM diameter in nm) were tested in this study. CD-1 mice were exposed to the ENM by oropharyngeal aspiration at a dose of 100 μg. Mouse lung tissue slices and alveolar macrophages were also exposed to the ENM at concentrations of 22–132 and 3.1-100 μg/mL, respectively. Biomarkers of lung injury and inflammation were assessed at 4 and/or 24 hr post-exposure.

**Results:**

Small-sized ENM (SiO_2_ (10), CeO_2_ (23), but not TiO_2_ (10)) significantly elicited pro-inflammatory responses in mice (*in vivo*), suggesting that the observed toxicity in the lungs was dependent on size and chemical composition. Similarly, SiO_2_ (10) and/or CeO_2_ (23) were also more toxic in the lung tissue slices (*ex vivo*) and alveolar macrophages (*in vitro*) compared to other ENM. A similar pattern of inflammatory response (e.g., interleukin-6) was observed in both *ex vivo* and *in vitro* when a dose metric based on cell surface area (μg/cm^2^), but not culture medium volume (μg/mL) was employed.

**Conclusion:**

Exposure to ENM induced acute lung inflammatory effects in a size- and chemical composition-dependent manner. The cell culture and lung slice techniques provided similar profiles of effect and help bridge the gap in our understanding of *in vivo*, *ex vivo*, and *in vitro* toxicity outcomes.

**Electronic supplementary material:**

The online version of this article (doi:10.1186/s12951-014-0047-3) contains supplementary material, which is available to authorized users.

## Background

It is well recognized that nanotechnology has been rapidly growing and advancing over the past 10 years, and will continue to expand in numerous market sectors [1,2]. The advances in nanotechnology, however are accompanied by a need for better understanding of the exposure and toxicity of engineered nanomaterials (ENM) across their life-cycle. Moreover, the enormously diverse and applications of ENM (e.g., shapes, sizes, chemical and surface characteristics) are likely to result in a broad array of exposures and potentially adverse health outcomes. Thus, methods to evaluate and predict the toxicity of ENM are of considerable importance [3]. In particular, more information is needed on the interactions of ENM with lung tissue, since inhalation is a common exposure route and can also lead to potential systemic toxicity [1]. There is already substantial epidemiologic and toxicological evidence that inhaled ENM cause pulmonary effects (e.g., inflammation and/or edema) and/or extrapulmonary or systemic effects (e.g., thrombosis, dysrhythmias, and myocardial infarction) [4-7]. In general, nanotoxicology studies of the respiratory tract are performed with *in vivo* (e.g., mice and rats) or *in vitro* (e.g., airway/alveolar epithelial cells, macrophages, and dendritic cells) models. Because of the inherent anatomical complexity of the intact lung which is comprised of about 40 different cell types interpretation of toxicity of ENM in *in vitro* cell culture models is limited as they do not reflect the complex cell-cell contacts and cell-matrix interactions in the tissue. Moreover, despite the need for studying the toxicity of ENM *in vivo*, there is a growing concern that broad toxicity testing will increase the number of animals required. Therefore, developing credible alternative testing methods predictive of *in vivo* ENM toxicity are essential to screen potential hazards and health risks associated with inhalation exposures to these novel materials [2].

Here, we investigated pulmonary toxicity of five ENM: one silicon dioxide (SiO_2_), two cerium oxide (CeO_2_), and two titanium dioxide (TiO_2_) nanomaterials with different primary diameters. SiO_2_, CeO_2_, and TiO_2_ nanomaterials are already widely used in industrial processes and consumer products. CeO_2_ and TiO_2_ nanomaterials are the most abundantly produced metal oxide nanomaterials in the U.S. [8] and have been independently tested for adverse health effects *in vitro* and *in vivo*, but not in the same study design [9,10]. CeO_2_ nanomaterials are of interest because despite having the same crystalline form as the parent compound, the nano-sized material causes more oxidative stress as a result of subtle changes in their surface chemistry [11,12]. SiO_2_ nanomaterials (particularly the amorphous form), have also recently received attention in biomedical applications, yet their toxicity is not fully understood [13]. In the present study, we conducted acute toxicity tests in mice (*in vivo*), mouse lung tissue slices (*ex vivo*), and mouse alveolar macrophages (*in vitro*) to extrapolate, and compare the results between *ex vivo* or *in vitro* to *in vivo* toxicity testing approaches. Lung tissue slices have shown to preserve almost all cell types and interactions with the microenvironment (i.e., cell-cell or cell-matrix interactions), thus providing the most *in vivo*-like physiologically relevant response. Of all the different types of lung cells, alveolar macrophages are considered to be one of the first lines of a defense against inhaled particles and are primarily responsible for producing pro-inflammatory mediators [14]. The specific aims of this study were to determine the pulmonary toxicity and pro-inflammatory potential of ENM in mice, and compare these effects with the use of *ex vivo* lung slice and *in vitro* cell-based toxicity testing systems.

## Results

### Particle size distributions of ENM

Hydrodynamic diameters of ENM in the various solutions used in this study were determined by dynamic light scattering (Table 1). Diameters of all ENM suspended in water were greater than the specifications provided by the manufacturer, and were even larger when the materials were suspended in culture media. Of all the ENM studied, TiO_2_ (10) and SiO_2_ (10) were the most highly agglomerated. Since this clumping behavior controls the density of the ENM agglomerates in suspensions, we estimated the agglomerate density and presented the results in Table 1. SiO_2_ (10) had the lowest agglomeration density in any solution, indicating that this material was most likely to remain suspended in the solutions and less likely to interact with the cells. Agglomerated TiO_2_ (200), on the other hand had the highest density which would promote settling and a greater potential to come in contact with the cells on the plate bottom.

**Table 1:** **Physicochemical properties of engineered nanomaterials (ENM)**

**Chemical**	**ID**	**Primary diameter** ^**a**^ **(nm)**	**Hydrodynamic diameter** ^**b**^ **(nm)**				**Surface area (m** ^**2**^ **/g)**	**Raw density** ^**e**^ **(g/cm** ^**3**^ **)**	**Equivalent primary diameter** ^**f**^ **(nm)**	**Agglomerate density** ^**g**^ **(g/cm** ^**3**^ **)**		**Crystal form**
			**Water**	**Saline**	**CM** ^**c**^	**CM** ^**c**^				**CM** ^**c**^ **(** ***ex vivo*** **)**	**CM** ^**c**^ **(** ***in vitro*** **)**	
				**(** ***in vivo*** **)**	**(** ***ex vivo*** **)**	**(** ***in vitro*** **)**						
SiO_2_	SiO_2_ (10)	5-15^a^	401 ± 13	574 ± 96	458 ± 66	342 ± 44	590-690^a^	2.65	3.54	1.06 ± 0.01	1.07 ± 0.01	Amorphous
CeO_2_	CeO_2_ (23)	15-30^a^	131 ± 55	269 ± 91	796 ± 46	432 ± 133	30-50^a^	7.22	20.79	1.49 ± 0.03	1.88 ± 0.45	Cerianite
CeO_2_	CeO_2_ (88)	70-105^a^	162 ± 60	239 ± 52	500 ± 38	220 ± 31	8-12^a^	7.22	83.16	2.78 ± 0.18	4.23 ± 0.58	Cerianite
TiO_2_	TiO_2_ (10)	10^a^	402 ± 16	739 ± 10	645 ± 3	660 ± 62	100-130^a^	3.90	12.33	1.19 ± 0.00	1.19 ± 0.02	Anatase
TiO_2_	TiO_2_ (200)	200^a^	387 ± 12	690 ± 29	493 ± 6	417 ± 22	6.99^d^	3.90	202.92	2.65 ± 0.03	2.86 ± 0.12	Anatase

^a^provided by the manufacturer.

^b^determined by dynamic light scattering and expressed as mean ± SEM of multiple measurements.

^c^CM: culture medium.

^d^obtained from Sanders et al. [15].

^e^obtained from the CRC Handbook of Chemistry and Physics [16].

^f^calculated from *equivalent primary diameter =6/(SSA × ρ)* [17,18], where *SSA* is specific surface area, and *ρ* is raw nanomaterial density.

^g^calculated from the Sterling equation [19], *agglomerate density = (1-(1-(d*_*H*_ */d*_*Eq*_ *)* ^*DF-3*^ *))ρ + (1-(d*_*H*_ */d*_*Eq*_ *)* ^*DF-3*^ *)ρ*_*media*_, where *d*_*H*_ is hydrodynamic diameter, *d*_*Eq*_ is equivalent primary diameter, *DF* is theoretical fractal dimension (assuming *DF* =2.3 [20]), *ρ* is raw nanomaterial density, and *ρ*_*media*_ is media density (assuming *ρ*_*media*_ = 1 g/cm^3^).

### Pulmonary inflammation responses *in vivo*

**Figure 1:**
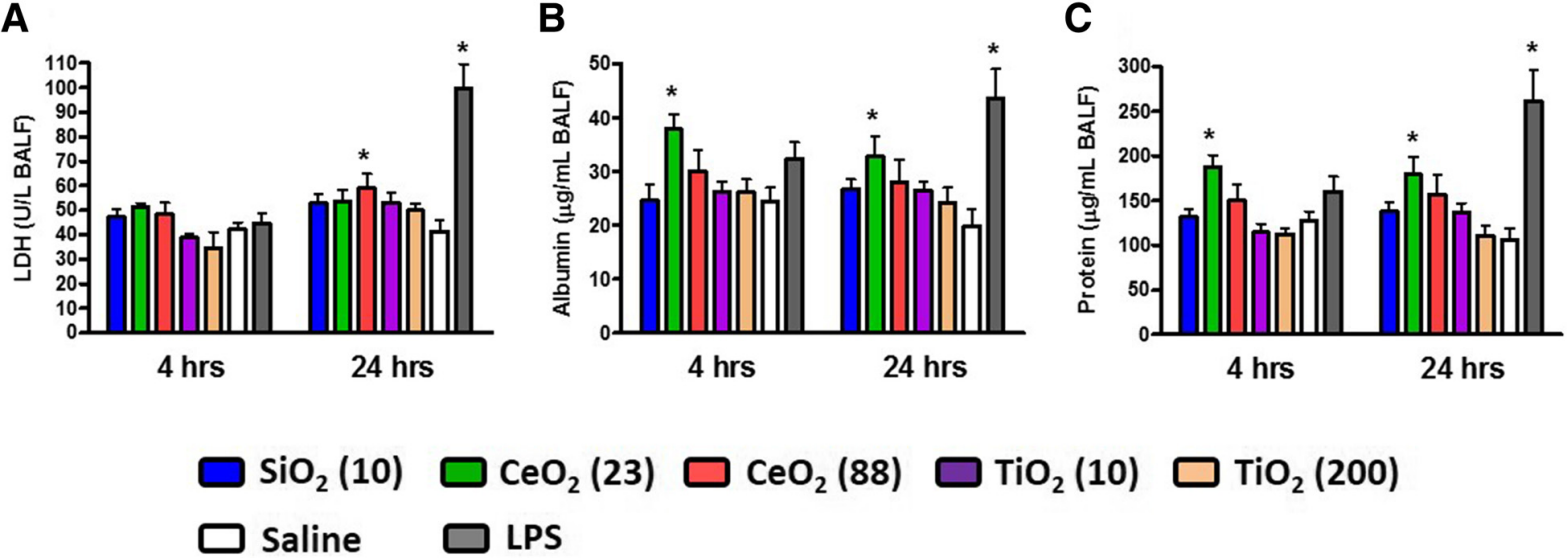
**Biochemical markers for lung injury and edema in BALF of mice at 4 hr and 24 hr post-exposure to ENM (100 μg) by oropharyngeal aspiration. (A)** LDH, **(B)** albumin, and **(C)** total protein concentrations in BALF. Data are means ± SEM (n =5-6 in each group). *p <0.05 compared with the saline-exposed negative control group from the same time point. Mice exposed to 2 μg of LPS served as a positive control.

**Figure 2:**
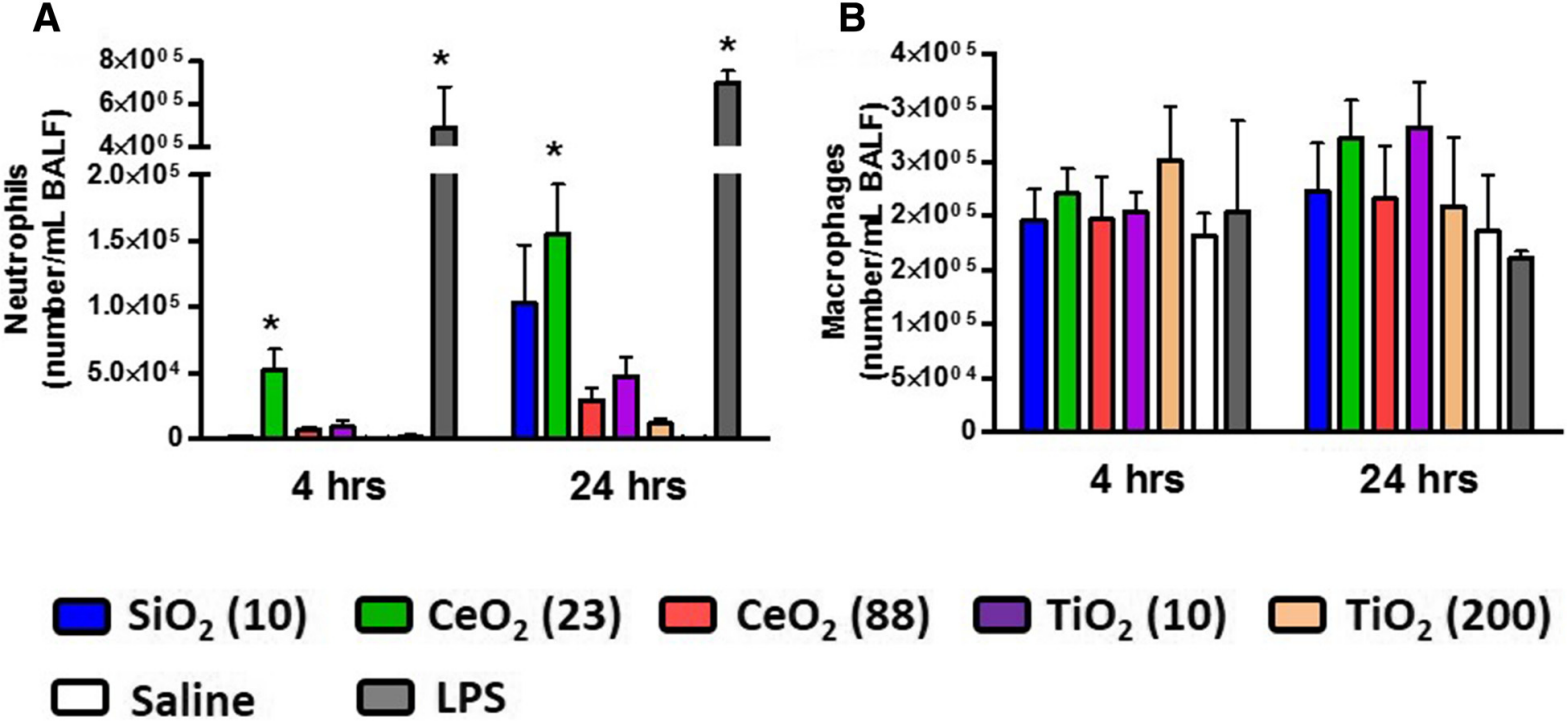
**Number of neutrophils and macrophages in BALF of mice at 4 hr and 24 hr post-exposure to ENM (100 μg) by oropharyngeal aspiration. (A)** neutrophils and **(B)** macrophages. Data are means ± SEM (n =5-6 in each group). *p <0.05 compared with the saline-exposed negative control group from the same time point. Mice exposed to 2 μg of LPS served as a positive control.

We monitored concentrations of lactate dehydrogenase (LDH) released into bronchoalveolar lavage fluid (BALF) at 4 hr and 24 hr post-exposure as a biomarker for lung cell injury. None of the ENM, except for CeO_2_ (88) (at 24 hr post-exposure), significantly increased the concentrations of LDH at any time point compared with saline control groups (Figure 1A). N-acetyl-β-D-glucosaminidase (NAG) and γ-glutamyl transferase (GGT) as biomarkers for lysosomal enzyme and oxidative stress, respectively, were also assessed and were unchanged for any of the ENM (data not shown). Concentrations of albumin and total protein in BALF from the CeO_2_ (23)-exposed groups were significantly increased at 4 hr and 24 hr post-exposure compared with saline-exposed groups, indicating that this material caused lung edema (Figure 1B and C). As a positive control, LPS increased LDH, albumin, and protein as expected, but did not affect NAG or GGT. The size- and composition-dependent toxicity of ENM was also seen in pulmonary inflammatory cells at 4 hr and 24 hr post-exposure (Figure 2). The CeO_2_ (23)-exposure groups significantly increased the number of neutrophils (18% and 34% at 4 hrs and 24 hrs, respectively), compared with saline controls. While LPS-exposure groups induced an even stronger neutrophil influx, no other ENM caused significant changes in the neutrophil number. The number of macrophages in BALF was unchanged by any treatment.

**Figure 3:**
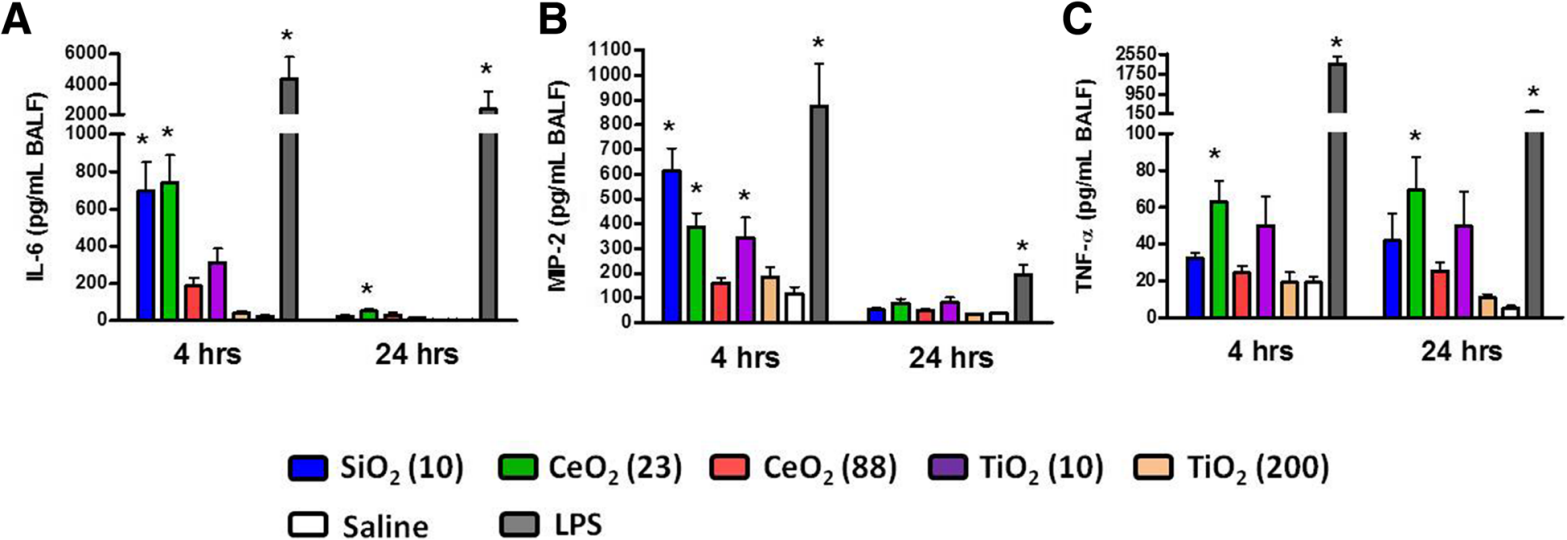
**Cytokine levels in BALF of mice at 4 hr and 24 hr post-exposure to ENM (100 μg) by oropharyngeal aspiration. (A)** IL-6, **(B)** MIP-2, and **(C)** TNF-α concentrations in BALF. Data are means ± SEM (n =5-6 in each group). *p <0.05 compared with the saline-exposed negative control group from the same time point. Mice exposed to 2 μg of LPS served as a positive control.

Concentrations of pro-inflammatory cytokines (interleukin-6 (IL-6), macrophage inhibitory protein-2 (MIP-2), and tumor necrosis factor-α (TNF-α)) were then monitored in BALF at both time-points (Figure 3). CeO_2_ (23) significantly increased the concentrations of all three cytokines at 4 hr post-exposure compared with saline control groups. SiO_2_ (10) significantly increased the concentrations of IL-6 and MIP-2 at 4 hr post-exposure, while TiO_2_ (10) but not the TiO_2_ (200) only increased the concentration of MIP-2. These data indicate that the small-sized ENM induced more acute lung inflammation than their larger counterparts, and that chemical composition of ENM was a more important determinant than their size. Based on the cytokine response results, toxicity ranking of ENM approximated CeO_2_ (23) ≈ SiO_2_ (10) > TiO_2_ (10) > CeO_2_ (88) > TiO_2_ (200). At 24 hr post-exposure, the cytokine concentrations decreased to saline control values except for CeO_2_ (23) which maintained elevated levels of IL-6 and TNF-α. Interestingly, the inflammation was not related to uptake of ENM in lung macrophages. The less active TiO_2_ (10) and TiO_2_ (200) were avidly taken up by lung macrophages at both time points compared with other ENM (Additional file 1: Figure S1). Finally, there were no significant changes in circulating white blood cells, red blood cells (RBCs) or RBC indices between the ENM-exposed mice and saline controls (data not shown).

### Pulmonary inflammation responses *ex vivo* and *in vitro*

**Figure 4:**
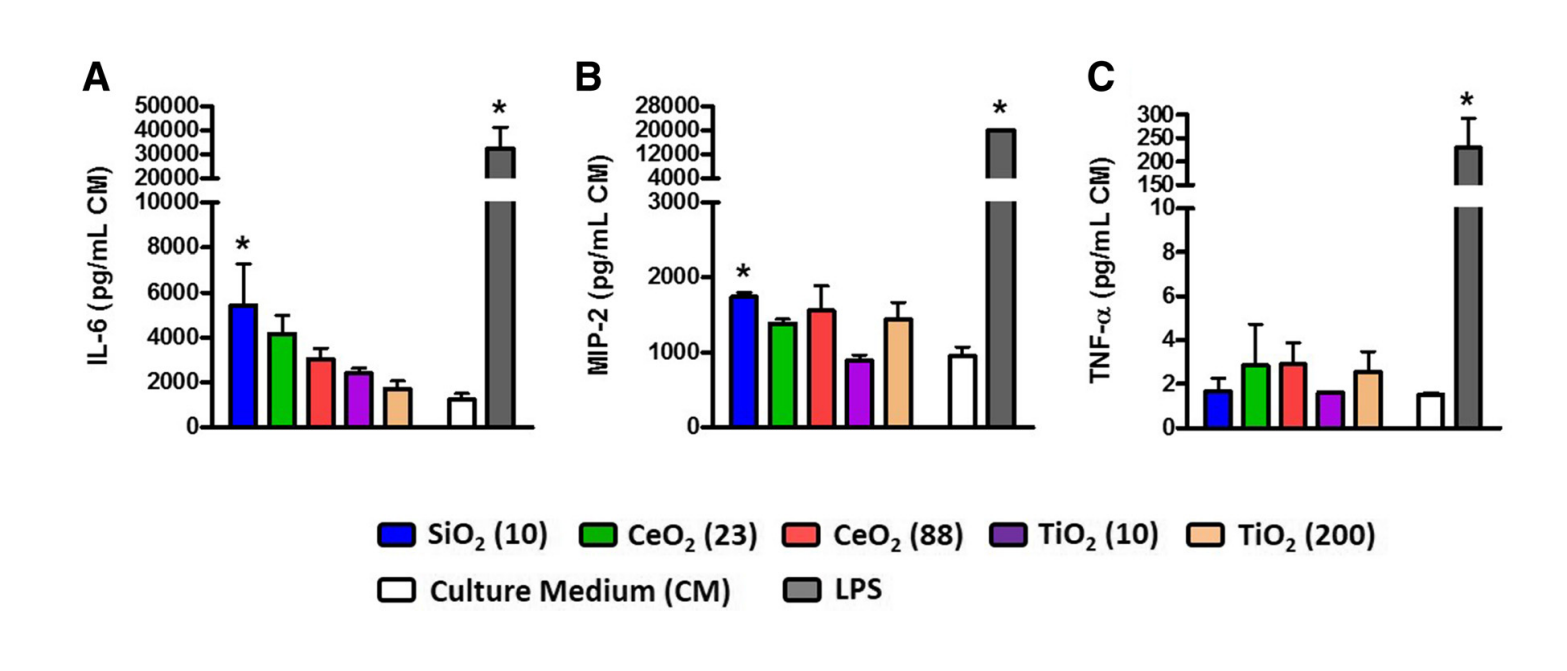
**Cytokine levels in lung tissue slices at 24 hr post-exposure to ENM (132 μg/mL). (A)** IL-6, **(B)** MIP-2, and **(C)** TNF-α concentrations in the culture medium (CM) from the lung tissue slices. Data are means ± SEM (n =3 in each group). *p <0.05 compared with CM-exposed negative control group. Lung tissue slices exposed to 87 ng/mL of LPS served as a positive control.

LDH, GGT, and NAG concentrations in the supernatants from the lung tissue slices at 24 hr post-exposure were unchanged at any of the concentrations tested (data not shown). Only the SiO_2_ (10) at the highest concentration (132 μg/mL) significantly increased the concentrations of IL-6 and MIP-2 compared with negative controls (Figure 4). CeO_2_ (23) also had increased IL-6 concentration but this was not statistically significant.

**Figure 5:**
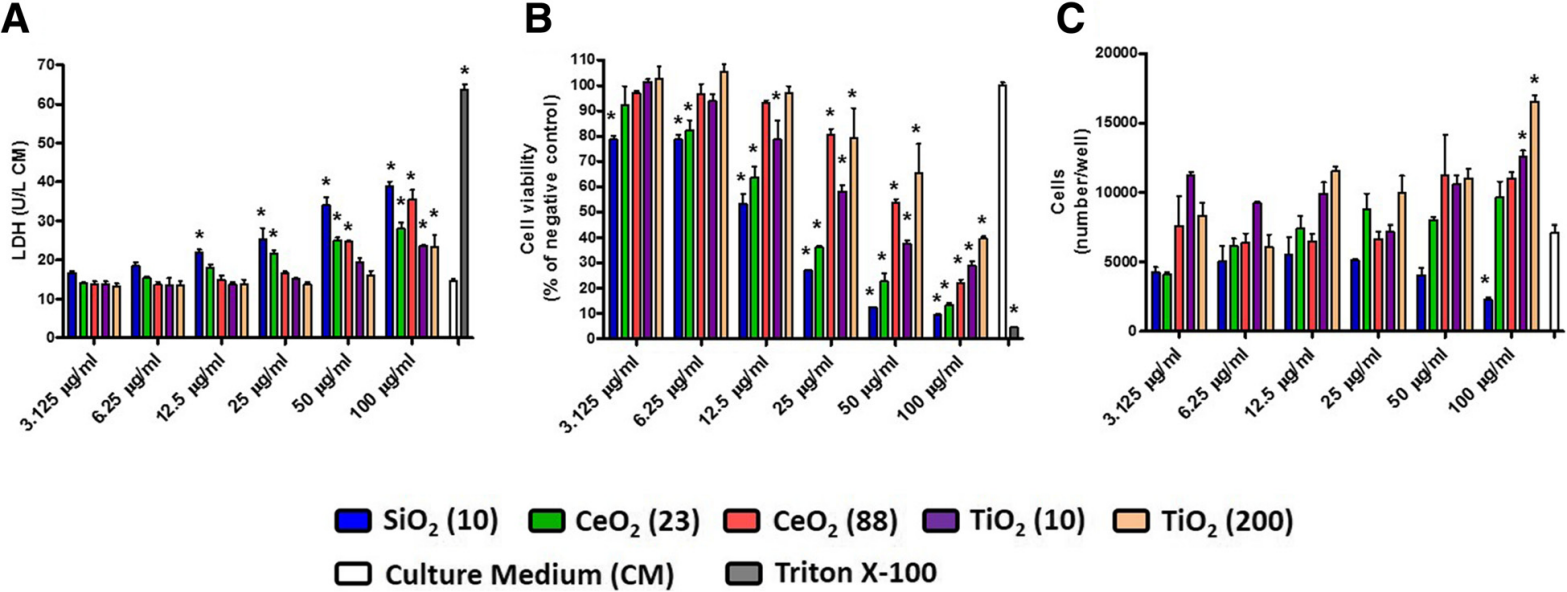
**Biochemical markers for cell membrane damage, viability, and proliferation in MH-S cells at 24 hr post-exposure to ENM (3.125-100 μg/mL). (A)** LDH concentrations in the culture medium (CM) from the MH-S cells, **(B)** cell viability based on metabolic activity of mitochondria, and **(C)** cell proliferation based on DNA content. Data are means ± SEM (n =3-6 in each group). *p <0.05 compared with CM-exposed negative control group. MH-S cells exposed to 1% Triton X-100 served as a positive control.

**Figure 6:**
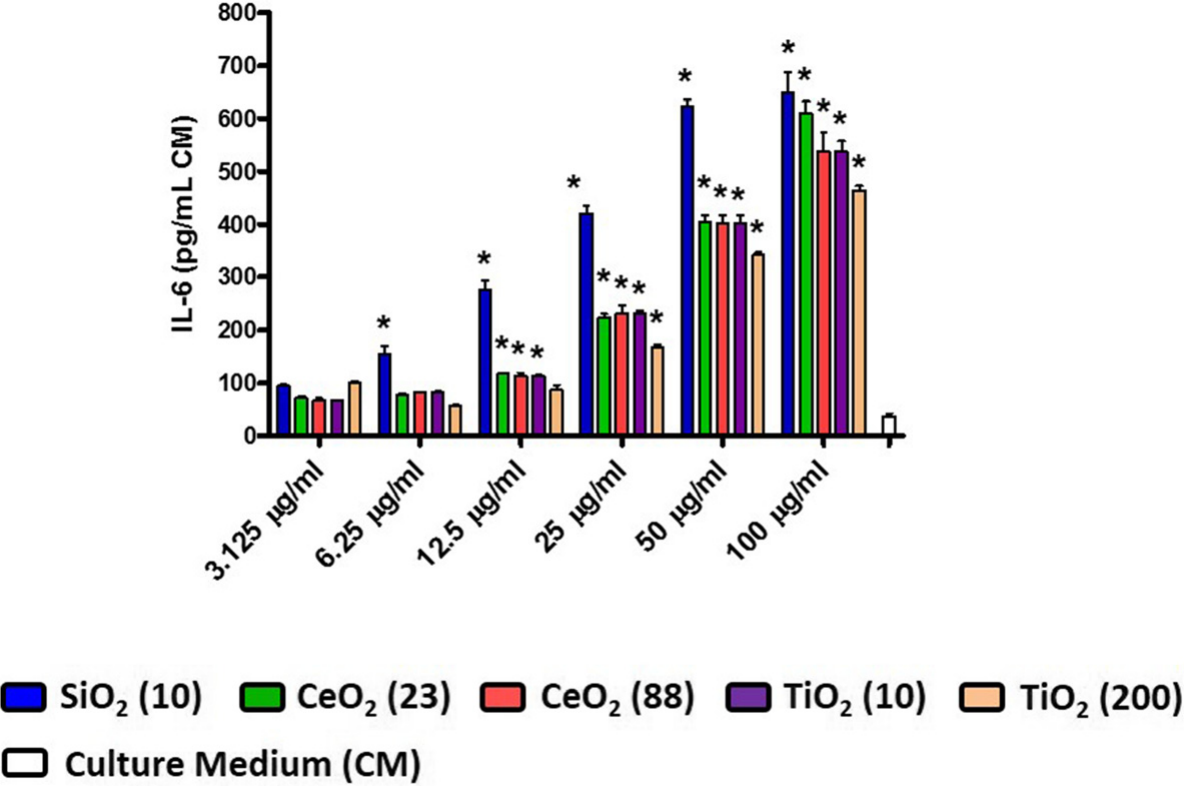
**Cytokine level in MH-S cells at 24 hr post-exposure to ENM (3.125-100 μg/mL).** Data are means ± SEM (n =3 in each group). *p <0.05 compared with CM-exposed negative control group.

Assessment of the cell culture supernatant from ENM-exposed MH-S cells at 24 hr post-exposure revealed that all ENM increased the LDH release in a dose-dependent manner (Figure 5A). SiO_2_ (10) and TiO_2_ (10 and 200) appeared to be more and less cytotoxic, respectively, however no apparent size-dependent effects (on cell membrane integrity) were observed. Half-maximal effective concentrations (EC_50_) for the cell membrane integrity of SiO_2_ (10), CeO_2_ (23), CeO_2_ (88), TiO_2_ (10), and TiO_2_ (200) were approximately 100, 295, 141, 330, and 384 μg/mL, respectively. Cell viability based on the metabolic activity of mitochondria was assessed at 24 hr post-exposure (Figure 5B). Similar to the LDH analysis data, we also observed dose-dependent effects of ENM. EC_50_ for the cell viability of SiO_2_ (10), CeO_2_ (23), CeO_2_ (88), TiO_2_ (10), and TiO_2_ (200) were approximately 13, 18, 55, 30, and 77 μg/mL, respectively (Additional file 2: Figure S2). Thus, toxicity ranking of ENM based on the EC_50_ for viability was in the order of SiO_2_ (10) > CeO_2_ (23) > TiO_2_ (10) > CeO_2_ (88) > TiO_2_ (200). Because the EC_50_ was much higher for LDH, this would indicate that the mitochondrial function was more sensitive to ENM exposure than cell membrane integrity. We also measured cell proliferation based on DNA content at 24 hr post-exposure and found that cell numbers did not significantly change in any of the ENM-exposed groups except at the high concentration exposure (Figure 5C). At 100 μg/mL concentration, SiO_2_ (10) significantly decreased MH-S cell numbers, while TiO_2_ (10) and TiO_2_ (200) significantly increased the cell numbers. Concentrations of pro-inflammatory cytokine, IL-6, in MH-S cells were measured at 24 hr post-exposure (Figure 6). SiO_2_ (10) induced more IL-6 production than other ENM which was in line with the IL-6 lung tissue slice response. To provide a more realistic comparison, we converted the nominal mass media concentration (i.e., μg/mL) to mass per unit cell (or tissue) surface area (i.e., μg/cm^2^) because lung tissue slices have a larger 3D surface area than the MH-S cells. Taking this into account the exposure dose of 132 μg/mL to the lung slice resulted in a dose of 4.7 μg/cm^2^. Therefore, the IL-6 responses in MH-S cells exposed to 12.5 μg/mL concentration (equivalent to 4.2 μg/cm^2^) was comparable to those in the lung tissue slices exposed to 132 μg/mL concentration (see the Materials and Methods section for a more detailed calculation).

## Discussion

While much work is being done to better understand the potential toxic effects of ENM on human health, it is still not clear which physico-chemical parameters of ENM are most important. Moreover, assessing (or screening) the toxic potential of emerging ENM is likely to increase the numbers of animals required, unless alternative methods are available that consistently reflect the *in vivo* biological effects. Here we utilized three different toxicity testing methods (mice, mouse lung tissue slices, and alveolar macrophages) to investigate the comparative toxicity of five ENM (SiO_2_ (10), CeO_2_ (23), CeO_2_ (88), TiO_2_ (10), and TiO_2_ (200)) and determine if the latter two techniques could predict effects seen in animals. We found, in all three different toxicity testing methods, that SiO_2_ (10) and/or CeO_2_ (23) had the highest activity on the basis of pro-inflammatory cytokine production. Importantly the mouse lung tissue slices and alveolar macrophages exhibited similar cytokine responses to the distinct ENM when the exposure dose metric was based on cell surface area.

### Size- and chemical composition-dependent lung toxicity of ENM in mice

Numerous studies of nanotoxicology have shown that toxicity of ENM is strongly influenced by two factors: 1) chemical toxicity based on the chemical composition of ENM, and 2) cellular stress caused by the physical properties of ENM [9]. In line with published reports, it was evident that only the smaller-sized ENM caused significant inflammatory effects on mouse lungs, and that the chemical composition was important since stronger effects were noted in SiO_2_ (10) and CeO_2_ (23) but not TiO_2_ (10). Interestingly TiO_2_ lung macrophage uptake was higher than the other ENM, despite displaying lower toxicity suggesting that the observed inflammatory responses were not dependent on phagocytosis. In support of this, a similar study demonstrated that nanomaterial toxicity was not correlated with particle uptake in the cells [21].

Although further studies are needed to understand the mechanism underlying lung toxicity of ENM, the data also suggest that there was no clear relationship between lung toxicity and degree of ENM agglomeration (i.e., hydrodynamic diameters). Agglomerates of ENM form in biological fluids by loose binding (e.g., van der Waals force) while primary diameters, and not hydrodynamic diameters influence toxicity. In support of this, other researchers have reported that nanoparticle trafficking across lung epithelial cells was correlated with primary diameters and not the hydrodynamic diameters of the agglomerated nanoparticles [22].

Numerous studies have reported that ENM of various crystalline forms and solubility cause varying degrees of lung injury and inflammation. It is generally accepted that insoluble ENM are far less active in producing cellular damage or injury as compared to (partially) soluble ENM of similar size [23-25], although insoluble ENM have the potential to remain in the lungs and other organs for a long. It also should be noted that while insoluble ENM may not be potent enough to cause cell damage, crystallinity of the ENM (e.g., amorphous or crystalline) might contribute to other toxicological properties [10,26]. In addition, insoluble ENM may cause oxidative stress and lung inflammation depending on their conduction band energy levels [27]. The ENM used in this study (SiO_2_, CeO_2_, and TiO_2_) were insoluble (or poorly soluble) in biological fluids and considered not to release free ions from the nanomaterials to the tissue or cells. Here, the cytokine responses induced by SiO_2_ (10) and CeO_2_ (23) were evident in mice at 4 hr post-exposure but receded to control levels at 24 hrs, indicating that the inflammatory response was transient. Others have reported sustained pro-inflammatory cytokine levels at 24 hrs after exposure to SiO_2_ (amorphous and 14 nm) albeit with 50 mg/kg which is ~15 times higher than the concentration used here [28]. While lung toxicity of SiO_2_ nanomaterials has been extensively studied [26], there are only a few reports of lung toxicity of CeO_2_ nanomaterials [9,29-32]. Moreover, these studies have mainly focused on long-term toxicity in mice or rats, demonstrating that intratracheal instillation or inhalation of CeO_2_ nanomaterials led to severe chronic lung inflammation for up to 28 day post-exposure. Although our findings were limited to the 24 hr time-course, we cannot rule out the possibility of further chronic inflammatory responses, particularly in light of human case studies which report development of lung disease in workers after repeated long-term exposure of CeO_2_ [33,34]. Similarly, our results showed that TiO_2_ nanomaterials did not cause significant lung inflammation in mice, consistent with recently published TiO_2_ toxicity findings performed through multiple interlaboratory comparisons [35].

### Comparing lung toxicity testing in mice to its alternatives

Efforts to reduce the number of animals in toxicity testing have resulted in the development of numerous *ex vivo* and *in vitro* toxicity test methods but the results are still conflicting. This inconsistency could be due to the fact that there are 1) a lack of overall consensus on the relevant dose metric for *in vivo* and *ex vivo/in vitro* studies and 2) inherent limitations to most *in vitro* models such as a lack of complex cell-cell interactions [36]. Here, the mouse lung tissue slices (*ex vivo*) and MH-S cells (*in vitro*) displayed a similar pattern of cytokine response on the basis of the mass per unit surface area of cell or tissue (μg/cm^2^) but not per unit volume of culture medium (μg/mL), suggesting that cell surface area should be considered in *in vitro* dosimetry when comparing toxicity endpoints from different systems.

It is well documented that nanomaterials form agglomerates in suspension and their fate (or behavior) is governed by different mass transport properties (sedimentation and/or diffusion), leading to differential exposures of nanomaterials to cells [17,18,20]. The nominal mass media concentration (μg/mL) in submerged cell-culture conditions assumes that the suspended nanomaterials are completely deposited on the cell surface which may not be always true for all nanomaterials in suspension and may result in misinterpretation of biological response data [37]. In the present study the density of agglomerated ENM in suspension (which influences delivered dose) was associated with the resultant cellular responses *ex vivo* and *in vitro* [17,18,20]. Notably, if the agglomerate density approached the density of the culture medium, the nanomaterials were more likely to remain suspended in the medium (i.e., low delivered dose), leading to a reduced exposure and diminution of biological responses to the nanomaterials [17,18]. In this regard, since the agglomerate densities of SiO_2_ (10), CeO_2_ (23) and TiO_2_ (10) *ex vivo* and *in vitro* were closer to the culture media compared to other ENM, it is likely that the toxic effects were underestimated. In other words, the cytokine responses *ex vivo* and *in vitro* would be expected to increase even more if the cells were exposed to the same delivered dose. Therefore, considering the behavior of ENM agglomerates in submerged cell culture systems (*ex vivo* or *in vitro*) may reduce the disparity between *in vitro* and *in vivo* nanotoxicology outcomes. However, there are limitations to be considered when interpreting *in vitro* cellular responses based on agglomerate density. If ENM are soluble in culture media, their agglomerate density will change over time. Moreover, as described above, in the case of *in vitro* ENM toxicity tests, agglomerations may result in an underestimation of toxicity outcomes (or ranking), while in the case of *in vivo* ENM toxicity tests (via intratracheal instillation or oropharyngeal aspiration technique), agglomeration may cause an overestimation of toxicity outcomes (or ranking) [38]. It is also worth noting that the agglomeration associated with ability of ENM to absorb biological components (e.g., ions, salts, and proteins) in the *in vitro* and *in vivo* system may differently overshadow ENM properties (e.g., chemistry and surface charge), leading to the inconsistent results (*in vitro* versus *in vivo*) [39].

As aforementioned, one of the major challenges faced in cell-based *in vitro* models is that intact lungs are comprised of about 40 different cell types, and *in vitro* models cannot wholly reflect the microenvironment of cell-cell and cell-matrix interactions. Here we utilized the lung tissue slice model which preserves the lung architecture with nearly all cell types. We have previously reported that mouse lung tissue slices incubated with size fractionated particulate matter from a wildfire event displayed similar cytokine responses to those observed in mice [40]. In line with this finding, the lung tissue slice system also showed similar pro-inflammatory responses to ENM as those seen in mice (i.e., pro-inflammatory effects of SiO_2_ (10) and CeO_2_ (23) but not TiO_2_ (10)). Taken together, the results provide further evidence for particle-mediated biological responses in lung tissue slices and the feasibility of this application to lung toxicity testing. Although several studies have demonstrated toxicity of ENM in lung tissue slices [41,42], this is the first report to our knowledge to compare responses to different size and types of ENM in both mice and mouse lung tissue slices. In addition, the rank order of ENM IL-6 production from the MH-S cells was the same as that observed in both the *ex vivo* and *in vivo* comparisons suggesting that lung macrophages play an important role in this response. In contrast, the response ranking for TNF-α (which is expressed at lower levels in lung macrophages compared to IL-6 [43]) was not the same, suggesting that this biomarker would not be a good readout across the three systems. It should be noted that lung epithelial cells and macrophages differ in pro-inflammatory responses following exposure to ENM [44,45] and that toxicity differs depending on the cell of origin [36], as demonstrated by the observation that cancerous cells are more toxic than their normal precursors.

## Conclusions

We conclude that small-sized ENM, SiO_2_ (10) and CeO_2_ (23) but not TiO_2_ (10), caused acute lung toxicity in mice (*in vivo*). CeO_2_ (23) had the strongest effect on cytokine (IL-6, TNF-α, and MIP-2) release, neutrophil recruitment, and increased protein into the mouse lungs, while the larger CeO_2_ (88) and TiO_2_ (200) were less potent, indicating that the effect was dependent on both size and chemical composition of ENM. The rank order of ENM toxicity from both lung tissue slices (*ex vivo*) and alveolar macrophages (*in vitro*) corresponded well to the ranking results from the mice (*in vivo*), suggesting that lung macrophages could replicate this effect. The similar profile of inflammatory response *ex vivo* and *in vitro* was most apparent when the exposure was based on mass per cell surface area. Although we demonstrated a relatively good correlation among the acute lung toxicity endpoints from three different testing methods, further studies are still needed that measure reversibility of effects or progression to long term toxicity. Nevertheless the results provide further evidence for the feasibility of replacing animal lung toxicity testing with cells or lung tissue slices, and provide information about the important parameters (e.g., agglomeration state and exposure dose metric) that will improve interpretation of ENM toxicity in biological systems.

## Materials and methods

### Experimental animals

Adult pathogen-free female CD-1 mice (~20-25 g and ~30-45 g body weights for pulmonary toxicity and lung tissue slice studies, respectively) purchased from Charles River Breeding Laboratories (Raleigh, NC). Mice were housed in groups of five in polycarbonate cages with hardwood chip bedding at the U.S. Environmental Protection Agency (EPA) Animal Care Facility accredited by the Association for Assessment and Accreditation of Laboratory Animal Care and were maintained on a 12-hour light to dark cycle at 22.3 ± 1.1°C temperature and 50 ± 10% humidity. Mice were given access to rodent chow and water *ad libitum* and were acclimated for at least 10 days before the study began. The studies were conducted after approval by the EPA Institutional Animal Care and Welfare Committee.

### Engineered nanomaterials (ENM)

Five ENM were used in this study and designated by their mean primary diameter provided by the manufacturer: SiO_2_ (10) (silicon dioxide with a primary diameter of 5–15 nm; amorphous; Sigma Aldrich (St. Louis, MO)), CeO_2_ (23) (cerium oxide with a primary diameter of 15–30 nm; cerianite; NanoAmor (Houston, TX)), CeO_2_ (88) (cerium oxide with a primary diameter of 70–105 nm; cerianite; Alfa Aesar (Ward Hill, MA)), TiO_2_ (10) (titanium dioxide with a primary diameter of 10 nm; anatase; Alfa Aesar), and TiO_2_ (200) (titanium dioxide with a primary diameter of 200 nm; anatase; Acros Organics (Fair Lawn, NJ)). The ENM were suspended in saline for *in vivo* and culture media (see below for further details) for *ex vivo* and *in vitro*, followed by sonication (Sonicator 4000; Misonix Sonicators, Newtown, CT) at 70–80 watts for 10 min and vortex mixing for 1 min to yield a stock solution at a concentration of 2 mg/mL. The ENM suspensions were stored at −80°C until toxicity testing. To explore the effect of solution chemistry on hydrodynamic diameters of ENM, dynamic light scattering (Zetasizer Nano ZS; Malvern Instruments, Malvern, UK) was used at 100 μg/mL ENM concentration in various solutions, such as distilled water, saline, and culture media. Further detailed physicochemical characteristics of ENM are presented in Table 1.

### *In vivo* toxicity of ENM

#### Mouse exposure to ENM

Oropharyngeal aspiration was performed on mice anesthetized in a small plexiglass box using vaporized anesthetic isofluorane, following a technique described previously [46]. Briefly, the tongue of the mouse was extended with forceps and 100 μg of ENM in 50 μL saline was pipetted into the oropharynx. Immediately, the nose of the mouse was then covered causing the liquid to be aspirated into the lungs. Similarly, a separate group of mice was instilled with 2 μg of lipopolysaccharide (LPS; *Escherichia coli* endotoxin; 011:B4 containing 10^6^ unit/mg material; Sigma) as a positive control to demonstrate maximal responsiveness to this well characterized inflammatory agent while additional mice were instilled with 50 μL saline alone as a negative control.

#### Bronchoalveolar lavage and hematology

At 4 hr and 24 hr post-exposure, six mice from each treatment group were euthanized with 0.1 mL intraperitoneal injection of Euthasol (diluted 1:10 in saline; 390 mg pentobarbital sodium and 50 mg phenytoin/mL; Virbac AH, Inc., Fort Worth, TX), and blood was collected by cardiac puncture using a 1-mL syringe containing 17 μL sodium citrate to prevent coagulation. The trachea was then exposed, cannulated and secured with suture thread. The thorax was opened and the left mainstem bronchus was isolated and clamped with a microhemostat. The right lung lobes were lavaged three times with a single volume of warmed Hanks balanced salt solution (HBSS; 35 mL/kg mouse). The recovered bronchoalveolar lavage fluid (BALF) was centrifuged at 800x*g* for 10 min at 4°C and the supernatant was stored at both 4°C (for biochemical analysis) and −80°C (for cytokine analysis). The pelleted cells were resuspended in 1 mL HBSS (Sigma). Total BALF cell count of each mouse was obtained by a Coulter counter (Coulter Co., Miami, FL). Additionally, 200 μL resuspended cells were centrifuged in duplicate onto slides using a Cytospin (Shandon, Pittsburgh, PA) and subsequently stained with Diff-Quik solution (American Scientific Products, McGraw Park, PA) for enumeration of macrophages and neutrophils with at least 200 cells counted from each slide. Hematology values including total white blood cells, total red blood cells, hemoglobin, hematocrit, mean corpuscular volume, mean corpuscular hemoglobin concentration, and platelets were measured using a Coulter AcT 10 Hematology Analyzer (Beckman Coulter Inc., Miami, FL).

#### Biochemical and cytokine analyses

Concentrations of lactate dehydrogenase (LDH) and γ-glutamyl transferase (GGT) were determined using commercially available kits (Thermo Scientific, Middletown, VA). Albumin and total protein concentrations were measured by the SPQ test system (DiaSorin, Stillwater, MN) and the Coomassie plus protein assay (Pierce Chemical, Rockford, IL) with a standard curve prepared with bovine serum albumin (Sigma), respectively. Activity of N-acetyl-β-D-glucoaminidase (NAG) was determined using a NAG assay kit (Roche Applied Science, Indianapolis, IN). All biochemical assays were modified for use on the KONELAB 30 clinical chemistry spectrophotometer analyzer (Thermo Clinical Lab Systems, Espoo, Finland) as described previously [46]. Concentrations of tumor necrosis factor-α (TNF-α), interleukin-6 (IL-6) and macrophage inhibitory protein-2 (MIP-2) in BALF were determined using commercial multiplexed fluorescent bead-based immunoassays (Milliplex Map Kit, Millpore Co., Billerica, MA) measured by a Luminex 100 (Luminex Co., Austin, TX) following the manufacturer’s protocol. The limits of detection (LOD) of each cytokine were 6.27, 3.28 and 29.14 pg/mL for TNF-α, IL-6 and MIP-2, respectively, and all values below these lowest values were replaced with a fixed value of one-half of the LOD value.

### *Ex vivo* toxicity of ENM

#### Mouse lung tissue slice preparation and incubation

Lung tissue slices were prepared as previously described [40]. Briefly, mice were euthanized with 0.1 mL intraperitoneal injection of Euthasol (diluted 1:10 in saline; Virbac AH, Inc.). The trachea was exposed and cannulated using a 20G luer stub adapter (Instech Solomon, Plymouth Meeting, PA). The lungs were filled with 1.5% (w/v) low-melting agarose (Sigma) in minimum essential medium (MEM; Simga) at 37°C. The lungs were rinsed with the ice-cold slicing buffer solution (Earle’s balanced salt solution (Sigma) supplemented with 15 mM N-(2-hydroxyethyl)piperazine-N’-(2-ethanesulfonic acid) hemisodium salt (HEPES; Sigma)) and removed from the mouse. The lungs were transferred into the ice-cold slicing buffer solution to further solidify the agarose and then the lung lobes were separated using a surgical blade, and the lung tissue cores (8 mm diameter) were prepared using a tissue coring tool (Alabama Research and Development, Munford, AL). Tissue cores were cut into 350 μm thick slices in the ice-cold slicing buffer solution using a specialized vibratome (OTS 5000, FHC Inc., Bowdoinham, ME). The lung tissue slices were then incubated in the wash buffer solution (Dulbecco’s modified eagle’s medium/nutrient mixture F-12 Ham (Sigma) supplemented with 100 units/mL penicillin (Sigma) and 100 μg/mL streptomycin (Sigma)) under cell culture conditions for 4 hrs. The lung tissue slices were then transferred into a tissue culture treated polystyrene 48-well plate (Corning Inc., Corning, NY) and cultured in the slice incubation medium (the wash buffer solution supplemented with 200 mM L-glutamine (Sigma), 0.1 mM MEM non-essential amino acids (Sigma) and 15 mM HEPES) for up to 6 days at 37°C in a humidified atmosphere of 5% CO_2_ and 95% air. The lung tissue slices received fresh media every day.

#### Mouse lung tissue slice exposure to ENM

Reconstituted ENM suspensions were sonicated for 2 min, vortexed for 1 min and further diluted with the slice incubation medium to achieve final concentrations of 22, 44, 66, and 132 μg/mL. On day 2 of culture, lung tissue slices were exposed to the ENM for 24 hrs. The initial concentration of 22 μg/mL (total volume of 0.5 mL, therefore of 11 μg of ENM per lung slice) was estimated to be five times higher than the *in vivo* exposure dose used in this study. If it is assumed that the lung surface area of a 20 g mouse is ~650 cm^2^, 1 cm^3^ mouse lung tissue has ~800 cm^2^ lung surface area, and 100% of oropharyngeal instilled ENM is delivered to the lungs, 100 μg of ENM dose in a mouse (~650 cm^2^ lung surface area) is equivalent to 2.2 μg of ENM dose in a mouse lung slice (~14 cm^2^ lung slice surface area) [47]. Moreover, if it is assumed that the lung slice surface area is ~14 cm^2^, the exposure doses of 22, 44, 66, and 132 μg/mL are equivalent to the doses of 0.79, 1.6, 2.3, and 4.7 μg/cm^2^, respectively. Mouse lung tissue slices were exposed to 87 ng/mL LPS which was an equivalent concentration *in vivo* and served as a positive control. Mouse lung tissue slices exposed to the culture medium alone served as a negative control. At 24 hr post-exposure, lung slice culture fluids were collected, centrifuged at 10,000x*g* for 5 min, and culture supernatants were stored at both 4°C (for extracellular biochemical analysis) and −80°C (for cytokine analysis). Subsequently, mouse lung tissue slices were homogenized using a tissue homogenizer in a lysis buffer solution containing 0.5% Triton X-100, 150 mM NaCl, 15 mM Tris–HCl (pH 7.4), 1 mM CaCl_2_ and 1 mM MgCl_2_ [48]. Homogenates were then centrifuged at 10,000x*g* for 10 min and supernatants were stored at −80°C (for intracellular biochemical analysis).

#### Biochemical and cytokine analyses

Similar to the *in vivo* lung inflammation analyses described above, the supernatants of tissue culture fluids and tissue homogenates after exposure to ENM were used to determine the extracellular (LDH and NAG) and intracellular (GGT) biochemical analyses as well as cytokine analysis (IL-6, MIP-2, and TNF-α). Biochemical and pro-inflammatory cytokine analyses were performed using a KONELAB 30 clinical chemistry spectrophotometer analyzer (Thermo Clinical Lab Systems) and multiplexed fluorescent bead-based immunoassays (Milliplex Map Kit) measured by the Luminex 100 (Luminex Co).

### *In vitro* toxicity of ENM

#### Alveolar macrophage cell culture

The murine alveolar macrophages (MH-S) cells were purchased from ATCC (CRL2019, Manassas, VA) and grown in the following culture medium: RPMI 1640 (Sigma) supplemented with 5% fetal bovine albumin (FBS; Sigma) and 100 units/mL penicillin (Sigma) and 100 μg/mL streptomycin (Sigma) at 37°C in a humidified atmosphere of 5% CO_2_ and 95% air. MH-S cells at passage 11 yielded 2.4 - 2.9 *×* 10^6^ cells/mL and were seeded at 3,000 cells per well of a 96-well culture plate.

#### Alveolar macrophage cell exposure to ENM

After 3 days in culture, MH-S cells were exposed to ENM at final concentrations of 3.125, 6.25, 12.5, 25, 50, and 100 μg/mL in the culture medium for 24 hrs. This exposure dose can be converted to the dose based on cell surface area (assuming MH-S cell surface area is 0.3 cm^2^). Thus, the exposure doses of 3.125, 6.25, 12.5, 25, 50, and 100 μg/mL are equivalent to the doses of 1.0, 2.1, 4.2, 8.3, 16.7, and 33.3 μg/cm^2^, respectively. MH-S cells exposed to the culture medium alone served as a negative control and 1% Triton X-100 at 37°C served as a positive control.

#### Biochemical and cytokine analyses

After the cells exposed to ENM, the plate was centrifuged at 400xg for 5 min, followed by collection of supernatants to analyze LDH concentrations. The supernatants were also used to determine cytokine production (IL-6). The MH-S cells after centrifugation were then used to evaluate cell proliferation (CyQuant assay; Invitrogen, Eugene, OR). Viability of the MH-S cells exposed to ENM was tested by measuring enzymatic activity based on the cellular cleavage of water-soluble tetrazolium salt (WST-1) to formazan in the cells using a WST-1 assay kit (Roche Applied Science). Biochemical and pro-inflammatory cytokine analyses in this study were also performed using a KONELAB 30 clinical chemistry spectrophotometer analyzer (Thermo Clinical Lab Systems) and multiplexed fluorescent bead-based immunoassays (Milliplex Map Kit) measured by the Luminex 100 (Luminex Co).

### Statistical analysis

Data were expressed as means ± the standard error of the mean (SEM). The results of the ENM-exposed groups were compared to those of the negative control group. Statistical comparison was performed by one-way analysis of variance (ANOVA) with the Newman-Keuls post-hoc test. Statistical analyses were performed using commercial software (GraphPad Prism 6.04, GraphPad Software, Inc., San Diego, CA). If the data did not meet the ANOVA assumptions of either normality or equal variances (Levene’s test; *p* >0.05), the data were transformed. Subsequent to the transformation, the data were checked for requirement compliance and if acceptable, ANOVA proceeded. The statistical significance level was assigned at a probability value of *p* <0.05.

## Additional files

Additional file 1: Figure S1. Representative BAL cell images at 4 hr and 24 hr post-exposure to ENM. Red arrows indicate ENP uptakes in alveolar macrophages. Original magnification (20x), inset (40x). Additional file 2: Figure S2. Dose–response curves to determine EC_50_ values for the MH-S cells from WST-1 assay data.

## Abbreviations

ANOVA: Analysis of variance
BALF: Bronchoalveolar lavage fluid
CeO_2_ (23): Cerium oxide with a primary diameter of 15–30 nm
CeO_2_ (88): Cerium oxide with a primary diameter of 70–105 nm
CM: Culture medium
EC_50_: Half-maximal effective concentrations
ENM: Engineered nanomaterials
GGT: γ-glutamyl transferase
HBSS: Hanks balanced salt solution
HEPES: N-(2-hydroxyethyl)piperazine-N’-(2-ethanesulfonic acid) hemisodium salt
IL-6: Interleukin-6
LDH: Lactate dehydrogenase
LPS: Lipopolysaccharide
MEM: Minimum essential medium
MIP-2: Macrophage inhibitory protein-2
NAG: N-acetyl-β-D-glucoaminidase
RBC: Red blood cells
SEM: Standard error of the mean
SiO_2_ (10): Silicon dioxide with a primary diameter of 5–15 nm
TiO_2_ (10): Titanium dioxide with a primary diameter of 10 nm
TiO_2_ (200): Titanium dioxide with a primary diameter of 200 nm
TNF-α: Tumor necrosis factor-α
WST-1: Water-soluble tetrazolium salt
