# Supplementary figures and images for: Comparative lung toxicity of engineered nanomaterials utilizing in vitro, ex vivo and in vivo approaches
Source: J Nanobiotechnology. 2014 Nov 26;12:47. doi: 10.1186/s12951-014-0047-3 (PMC4262188; doi:10.1186/s12951-014-0047-3)

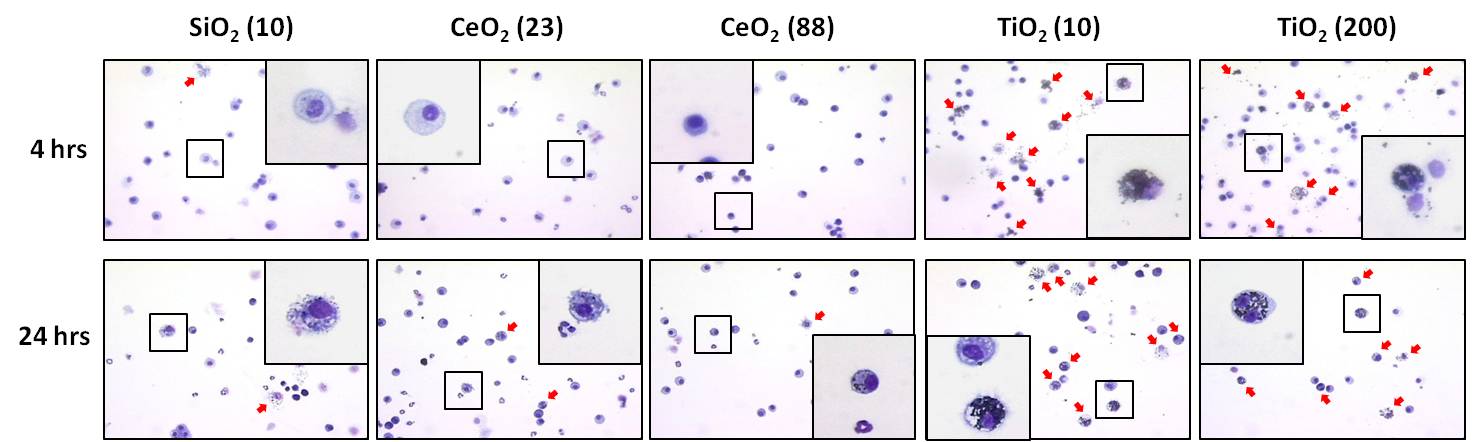

Supplement: Additional file 1: Figure S1. — Representative BAL cell images at 4 hr and 24 hr post-exposure to ENM. Red arrows indicate ENP uptakes in alveolar macrophages. Original magnification (20x), inset (40x). [file 12951_2014_47_MOESM1_ESM.jpeg]

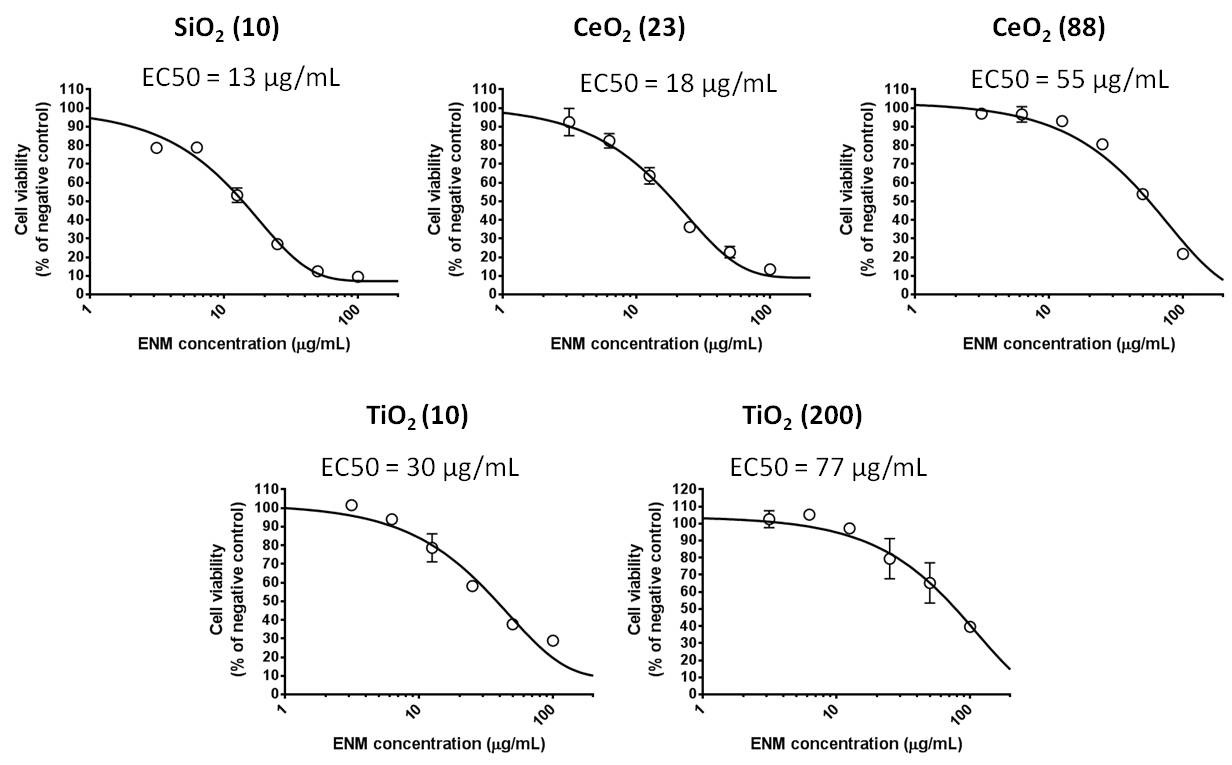

Supplement: Additional file 2: Figure S2. — Dose–response curves to determine EC50 values for the MH-S cells from WST-1 assay data. [file 12951_2014_47_MOESM2_ESM.jpeg]
